# Trigeminal Ganglion Neurons of Mice Show Intracellular Chloride Accumulation and Chloride-Dependent Amplification of Capsaicin-Induced Responses

**DOI:** 10.1371/journal.pone.0048005

**Published:** 2012-11-08

**Authors:** Nicole Schöbel, Debbie Radtke, Matthias Lübbert, Günter Gisselmann, Ramona Lehmann, Annika Cichy, Benjamin S. P. Schreiner, Janine Altmüller, Alan C. Spector, Jennifer Spehr, Hanns Hatt, Christian H. Wetzel

**Affiliations:** 1 Department of Cell Physiology, Ruhr-University Bochum, Bochum, Germany; 2 Leibniz Research Centre for Working Environment and Human Factors, University of Dortmund, Dortmund, Germany; 3 Institute for Biology II, Rheinisch-Westfaelische Technische Hochschule -Aachen University, Aachen, Germany; 4 Cologne Center for Genomics, University of Cologne, Cologne, Germany; 5 Department of Psychology, Florida State University, Tallahassee, Florida, United States of America; 6 Molecular Neurosciences, Department of Psychiatry and Psychotherapy, University of Regensburg, Regensburg, Germany; Monell Chemical Senses Center, United States of America

## Abstract

Intracellular Cl^−^ concentrations ([Cl^−^]_i_) of sensory neurons regulate signal transmission and signal amplification. In dorsal root ganglion (DRG) and olfactory sensory neurons (OSNs), Cl^−^ is accumulated by the Na^+^-K^+^-2Cl^−^ cotransporter 1 (NKCC1), resulting in a [Cl^−^]_i_ above electrochemical equilibrium and a depolarizing Cl^−^ efflux upon Cl^−^ channel opening. Here, we investigate the [Cl^−^]_i_ and function of Cl^−^ in primary sensory neurons of trigeminal ganglia (TG) of wild type (WT) and NKCC1^−/−^ mice using pharmacological and imaging approaches, patch-clamping, as well as behavioral testing. The [Cl^−^]_i_ of WT TG neurons indicated active NKCC1-dependent Cl^−^ accumulation. Gamma-aminobutyric acid (GABA)_A_ receptor activation induced a reduction of [Cl^−^]_i_ as well as Ca^2+^ transients in a corresponding fraction of TG neurons. Ca^2+^ transients were sensitive to inhibition of NKCC1 and voltage-gated Ca^2+^ channels (VGCCs). Ca^2+^ responses induced by capsaicin, a prototypical stimulus of transient receptor potential vanilloid subfamily member-1 (TRPV1) were diminished in NKCC1^−/−^ TG neurons, but elevated under conditions of a lowered [Cl^−^]_o_ suggesting a Cl^−^-dependent amplification of capsaicin-induced responses. Using next generation sequencing (NGS), we found expression of different Ca^2+^-activated Cl^−^ channels (CaCCs) in TGs of mice. Pharmacological inhibition of CaCCs reduced the amplitude of capsaicin-induced responses of TG neurons in Ca^2+^ imaging and electrophysiological recordings. In a behavioral paradigm, NKCC1^−/−^ mice showed less avoidance of the aversive stimulus capsaicin. In summary, our results strongly argue for a Ca^2+^-activated Cl^−^-dependent signal amplification mechanism in TG neurons that requires intracellular Cl^−^ accumulation by NKCC1 and the activation of CaCCs.

## Introduction

Cationic currents crucially generate neuronal excitation and are typically considered to be critical in stimulus detection and interneuronal communication. Nevertheless, anion homeostasis, in particular the regulation of intracellular Cl^−^ levels, contributes to neuronal excitation and signal transmission. For instance, the role of Cl^−^ in the regulation of signal transmission between dorsal root ganglion (DRG) neurons and second order neurons of the dorsal horn is under ongoing investigation [1]–[3]. In neurons of the developing central nervous system, the Na^+^-K^+^-2Cl^−^ cotransporter 1 (NKCC1) is crucial for the maintenance of elevated [Cl^−^]_i_ that is vital for neuronal survival and development [4]. In peripheral sensory neurons, namely frog DRG, the first description of NKCC1 was given by Alvarez-Leefmans and co-workers in 1988 [5]. As a consequence of NKCC1 transporter activity, central terminals of rat DRG neurons maintain a [Cl^−^]_i_ above its electrochemical equilibrium [6]. Therefore, the activation of presynaptic gamma-aminobutyric acid (GABA)_A_ receptors leads to primary afferent depolarization (PAD) that suppresses presynaptic output [2], [7] by inactivating voltage-gated channels and/or shunting incoming excitation [8], [9]. Pathological conditions can cause a further increase of [Cl^−^]_i_ by phosphorylation, recruitment, or upregulation of NKCC1 gene expression [10]–[14]. Higher [Cl^−^]_i_ in the afferent generates vigorous depolarization upon Cl^−^ efflux through GABA_A_ receptors. Block of NKCC1 by bumetanide, piretanide, or furosemide was shown to inhibit different nociceptive modalities, such as itch, injury-induced pain, and dorsal root reflexes in several studies [15]–[17].

The NKCC1^−/−^ mouse model has been generated in two laboratories [18], [19]. The animals have a complex phenotype involving complete deafness, male infertility, growth retardation, decreased blood pressure, as well as balance and motor deficits (shaker/waltzer phenotype) [20]. Interestingly, they also appeared to be less pain sensitive in a hot plate test [21], pointing out the importance of Cl^−^ homeostasis for determining the sensitivity of the entire somatosensory system.

Apart from the somatosensory system, Cl^−^ homeostasis is crucial for olfactory sensory neuron (OSN) function. OSNs display high [Cl^−^]_i_ as a result of NKCC1 activity [22]–[26]. In OSNs, a Ca^2+^-activated Cl^−^ current contributes to depolarization in the course of the odor-induced signal transduction cascade [22], [23], [27]–[29]. Tmem16b has been postulated as the functional Ca^2+^-activated Cl^−^ channel (CaCC) mediating the Ca^2+^-activated Cl^−^ current in OSNs [30]–[32]. This Cl^−^ efflux is thought to significantly contribute to the depolarization of the sensory neuron. Yang *et al*. proposed the new name Anoctamin (Ano) for the Tmem16 family referring to their anion conducting properties (anion) and the eight predicted transmembrane domains (octa  = 8) [33]. Besides the members of the anoctamin/Tmem16 family, several other proteins were predicted as CaCCs, including bestrophins [34], CLCAs (chloride channel, calcium-activated) [35], [36], the CLC family [37], [38], and the tweety family channels [39]. CaCCs have also been identified in DRG neurons [40], [41]. Here, only a subset of neurons expresses CaCCs, suggesting a specific role of these channels. For example, CaCCs could be detected only in medium diameter (Ø 30–40 µM) sensory neurons in the mouse DRG [42]. Functionally, Ano1 is the best described CaCC in DRG neurons being involved in the bradykinin-mediated depolarization [43] and heat response [44]. However, a possible function of CaCCs in signal amplification in trigeminal ganglion (TG) neurons has not been described to date.

In the present study, we investigated the Cl^−^ homeostasis of TG neurons and the function of Cl^−^ in these neurons. We found a high [Cl^−^]_i_ in a large fraction of TG neurons that depended on NKCC1 function. Most TG neurons displayed GABA-induced Cl^−^ efflux and Ca^2+^ responses, indicating Cl^−^-dependent depolarization of the cells. Interestingly, capsaicin-induced responses were diminished in neurons from NKCC1^−/−^ mice, suggesting a Cl^−^-based amplification of capsaicin-induced responses. Using next generation sequencing (NGS), we detected high levels of CaCC transcripts in TG neurons. Beyond that, pharmacological block of CaCCs reduced the capsaicin-induced responses of the neurons. In a behavioral paradigm, NKCC1^−/−^ mice displayed a higher tolerance for capsaicin-adulterated drinking water than the wild type (WT). Taken together, our results show elevated [Cl^−^]_i_ in most TG neurons that may exert dual effects. First, Cl^−^ may be involved in the regulation of afferent output of TG neurons onto second order neurons via PAD. Second, stimulus-induced elevations of cytosolic Ca^2+^ may lead to a depolarizing Cl^−^ efflux via CaCCs which in turn amplifies the sensory neuron's receptor potential.

## Materials and Methods

### Ethics statement

All experiments involving animals were carried out in accordance with the European Union Community Council guidelines and approved by the competent state office of the Federal Land of Northrhine Westphalia (file number 87–51.04.2010.A180).

### Animals

CD1 mice were obtained from Charles River (Sulzfeld, Germany). NKCC1 knockout mice (NKCC1^−/−^) were generated by Prof. Dr. Gary E. Shull, University of Cincinnati [19] and kindly provided by Prof. Dr. med. Ursula Seidler, University of Hannover. Animals were offered normal laboratory chow and water ad libitum.

### RT-PCR

RNA from various murine tissues (CD1 mice) was isolated with the RNeasy Mini Kit (Qiagen, Hilden, Germany) according to the manufacturer's protocol including DNaseI digestion. cDNA was prepared with the iScript kit (Bio-Rad). For each individual PCR the equivalent of 20 ng total RNA was used. PCR was performed under standard conditions with BioTherm Taq-Polymerase (Genecraft, Köln, Germany) in an Eppendorf realplex2 PCR machine (35×: 59°C, 72°C, 94°C, 1 min each). PCR-primers were intron spanning: ANO1fw: 5′-GGG AAA CAG CTG ATC CAG AA-3′, ANO1rv: 5′-ACA AAC TTT TTG GCG TCC AG-3′, (fragment size: 299 bp); ANO8fw: 5′-CCT CGT CAA CAA CCT GAT T-3′, ANO8rv: 5′-CCT CGT CAA CAA CCT GAT T-3′, (fragment size: 242 bp); TTHY3fv: 5′-ACT GAG TGG GGA CAT TTT GC-3′, TTHY3rv: 5′-TGG AGA TTC ACT TCC GTT CC-3′, (fragment size: 213 bp); GAPDH (glyceraldehyde-3-phosphate dehydrogenase)fv: 5′-TGT GTC CGT CGT GGA TCT GA-3′, GAPDHrev: 5′-CCT GCT TCA CCA CCT TCT TGA-3′, ßActinfw: 5′-GCA AGC AGG AGT ACG ATG AG-3′, ßActinrv: 5′-CCA TGC CAA TGT TGT CTC TT-3′.

For genotyping from post mortem tissue of newborn animals, the Phire® Animal Tissue Direct PCR kit (Finnzyme, Finland) was used according to the instruction manual. Primers for the wt gene were: NKCC1-6-5: 5′-GGA ACA TTC CAT ACT TAT GAT AGA TG-3′ and NKCC1-6-3: 5′-CTC ACC TTT GCT TCC CAC TCC ATT CC-3′ (fragment size: 105 bp). Primers for the mutant gene were: NKCC1-6-5: 5′- GGA ACA TTC CAT ACT TAT GAT AGA TG-3′ and dNEO-PolyA: 5′ GAC AAT AGC AGG CAT GCT GG-3′ (fragment size: 156 bp).

### Transcriptome analysis

For transcriptome analysis RNA from pooled complete trigeminal ganglia, dorsal root ganglia, and olfactory epithelia from adult CD1 mice was prepared. Total RNA was isolated with the RNeasy Mini Kit (Qiagen) according to the manufacturer's protocol including DNaseI digestion. At the Cologne Center for Genomics Next Generation Sequencing unit, libraries for NGS sequencing were constructed from total RNA and subjected to DSN normalization. RNA-Seq was performed on the Illumina GA_IIx_ sequencing platform as single reads with 36-nucleotide length. We essentially analyzed the sequence data as described [45]. Raw sequence data was aligned to the mouse genome reference sequence (mm9) using the TopHat aligner. Thereby, we could map 37 Mio. or 52 Mio. reads for male and female olfactory epithelium (OE) and 33 Mio. or 31 Mio. reads for TG or DRG. Output BAM-files were sorted and indexed using the Samtools software package [46]. FPKM (fragments per kilobase of exon per million fragments mapped) values were subsequently calculated by the Cufflinks program using the mm9refseq reference transcriptome only regarding the subset of protein coding genes for FPKM calculation. For our current study, we extracted the values for calcium activated chloride channel genes and typical housekeeping genes out of this data set. A detailed, comprehensive description of the complete TG, DRG and OE transcriptomes will be given elsewhere. For comparison to chemosensory tissue, we reanalyzed already published raw RNA-seq data from brain, liver, muscle [47] and testis [48] in the same manner as our own data.

#### Primary culture of trigeminal ganglion neurons

The culture of TG neurons was performed as described previously [49], [50]. In brief, mice were decapitated, the skull was opened, the brain removed, and the thereby exposed ganglia were dissected. Ganglia were then washed in PBS^++^ and collected in ice cold Leibovitz medium (L15, Invitrogen). Ganglia were transferred to essential medium (MEM, Invitrogen) containing 0.025% collagenase (type IA, Sigma), minced and incubated for 45 min at 37°C at 6% CO_2_. After enzymatic digestion, the ganglia were triturated with fire polished glass pipettes of decreasing tip diameter. The resulting cell suspension was centrifuged for 5 minutes at 1,000 rpm and the pellet was resuspended in Dulbecco's modified eagle medium F-12 (DMEM/F-12, GlutaMAX, Invitrogen) supplemented with 10% fetal calf serum (FCS) and 1% penicillin/streptomycin. After passing through a 70 µm cell sieve (Falcon) to remove pieces of tissue that were not dissociated by trituration, the cell suspension (50 µl) was plated on plastic cell culture dishes for patch clamp or glass cover slips for imaging experiments, both coated with poly-L-lysine (Sigma). After one hour of settling, 2 ml DMEM/F-12+10% FCS +1% pen/strep were added to the seeded cells. Primary cultures of trigeminal ganglia were either obtained from mice aged postnatal day (P)0-5 (newborn) or >P60 (adult). In vitro experiments were performed on P0-5 TG neurons unless stated otherwise. All experiments involving primary TG cultures were carried out 1–3 days after preparation.

### Subcultivation of HEK293 cell

HEK293 cells were cultured in T75-cell culture flasks in Minimal Essential Medium (MEM) containing Earle's salts and 2 mM L-glutamine (Invitrogen, Karlsruhe, Germany) supplemented with 10% fetal bovine serum (Invitrogen, Karlsruhe, Germany) and 1% penicillin/streptomycin under humidified conditions (37°C, 95% air, 5% CO_2_). Cells were subcultivated two to three times a week.

### Transient transfection of HEK293 cells

For transient expression of mTRPV1, we used the recombinant mammalian expression plasmid pcDNA3 (Invitrogen, San Diego, USA) carrying the entire protein coding sequence. Semiconfluent cells were transiently transfected (1 µg cDNA per dish) in 35 mm cell culture petri dishes (Sarstedt, Numbrecht, Germany) using the CaP-precipitation method [51]. As a transfection marker we cotransfected green fluorescent protein (GFP) plasmid DNA at a concentration of one tenth of the concentration of the TRPV1 plasmid. Cells were analyzed 18–48 h after transfection.

### Chemicals

Chemicals were prepared as concentrated stock solutions in *aqua dest*., EtOH, or dimethyl sulfoxide (DMSO) and diluted to their final concentration with standard assay buffer, resulting in a maximal solvent concentration of 0.1%. Stock solutions were stored refrigerated or frozen according to the seller's instructions. Bicuculline, 4,4*′-*Diisothiocyanatostilbene*-*2,2'*-*disulfonic acid disodium salt (DIDS), GABA, Mg-ATP, mibefradil, Na-GTP, niflumic acid (NFA), nigericin, protocatechuic acid, and tributyltin were purchased from Sigma-Aldrich (Deisenhofen, D). Bumetanide and capsaicin were purchased from Calbiochem (La Jolla, CA, USA). ω–Conotoxin MVIIC and nimodipine were purchased from Tocris Bioscience (Bristol, UK).

#### The application system

For all patch clamp and imaging experiments, a custom-made, pressure-driven 7-in-1 application system connected to 7 individual, valve-controlled solution containers was used. Prior to an experiment, the miniaturized application cannula with a tip opening diameter of 0.4 mm was placed in close proximity to the cells within the visual field to enable precise substance application. Opposite to the application cannula, a suction cannula was installed to continuously remove applied solutions, thereby avoiding the accumulation of stimulus in the bath. Between the application of different test solutions, cells were constantly perfused with standard saline (140 mM NaCl, 5 mM KCl, 2 mM CaCl_2_, 1 mM MgCl_2_, 10 mM HEPES, pH 7.4 (NaOH/HCl), 310 mOsmol (glucose)) to avoid mechanical artifacts. Test substances were diluted in the respective saline. All solutions were applied at a flow rate of about 500 µl/min.

#### Patch clamp

Patch clamp experiments were conducted as previously described [49], [52]. Patch pipettes were pulled from borosilicate glass (1.17×1.50×111 mm with filament, Science Products, Hofheim, Germany) with a horizontal pipette puller (Zeitz Instr., Munich, Germany) and were fire polished to a tip resistance of 3–7 MΩ. All experiments were carried out in the whole cell mode at room temperature using a HEKA EPC7 amplifier. Capacities and series resistance were adjusted manually. Data were acquired using the Pulse software (HEKA, Lambrecht, Germany). The standard intracellular buffer was composed of: 140 mM KCl, 0.3 mM CaCl_2_, 1 mM EGTA, 10 mM HEPES, 2 mM Mg-ATP, and 1 mM Na-GTP. The solution was adjusted to pH 7.1 with KOH/HCl and to 290 mOsmol with glucose. The calculated free [Ca^2+^] was 100 nM.

### Chloride imaging

To measure changes of intracellular chloride concentrations ([Cl^−^]_i_) the quinolinium-based dye *N*-(ethoxycarbonylmethyl)-6-methoxyquinolinium bromide (MQAE, Invitrogen) was used. This dye is quenched rapidly (<1 ms) by Cl^−^ by collisional quenching [53]. As MQAE shows the highest fluorescence in the absence of Cl^−^, a decrease of the monitored fluorescence represents an increase in the [Cl^−^]_i_. For dye loading, cells on cover slips were incubated in cell medium containing 5 mM MQAE for 60 min at 37°C and 6% CO_2_. The cover slips were then transferred to a measuring chamber made of inert steel that was mounted on an inverted microscope (Axiovert 200, Zeiss) equipped with an oil-immersion objective (Zeiss, Fluar 40x, nA 1.30). MQAE was excited at 350 nm with a monochromator (Polychrom V, Till Photonics, Gräfelfing, Germany) and cell images were captured with a SensiCam camera (pco imaging, Germany). The emitted light was measured at 460 nm using a beam splitter (T-400 LP, Chroma) and band pass filter (ET-460/50 nm, Chroma).

Cells were excited for 60 ms every 500 ms for the stimulation protocol and every 15 s for the calibration protocol. Changes of [Cl^−^]_i_ were calculated as ΔF with
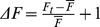
where F_t_ is the fluorescence at point of time *t* and 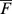 is the mean fluorescence of the first ten data points. Imaging data were acquired using the TILLvisION software (Till Photonics).

Control experiments using the parameters described above did not show any significant loss of fluorescence intensity over 10 min for the stimulation protocol and 30 min for the calibration protocol, respectively, so no bleaching effects had to be taken into account for the calculation.

For the calibration of MQAE fluorescence for [Cl^−^]_i_ the “double-ionophore” technique was used [54], [55]. The Cl^−^/OH^−^ antiporter tributyltin (Sigma-Aldrich, Germany) and the K^+^/H^+^ ionophore nigericin (Sigma-Aldrich, Germany) were added to solutions of defined chloride concentrations (0, 20, 40, and 60 mM). The calibration solution contained: 5 µM nigericin, 10 µM tributyltin, 10 mM HEPES, 150 mM K^+^, and variable amounts of methanesulfonic acid and Cl^−^ (depending on the desired final [Cl^−^] of the solution). Osmolarity was adjusted to 310 mOsmol with glucose.

Under steady state conditions the cytosolic [Cl^−^] is assumed to be equal to the corresponding calibration solution. The cells were exposed to the different solutions until a steady state condition was achieved (approx. 10 min). [Cl^−^]_i_ was calculated using the Stern-Volmer-equation:
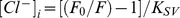
where F_0_ is the mean fluorescence at 0 mM chloride, F is the mean fluorescence intensity at a steady state condition in a calibration solution, and Ksv is the Stern-Volmer constant. Ksv was calculated for each cell, the average Ksv for all measured cells was 14.4±3.3 M^−1^. The Nernst Equation was used for calculations of E_Cl_. The equation was rearranged for calculations of the [Cl^−^]_i_ required to generate Cl^−^ efflux from TG neurons (see example below):



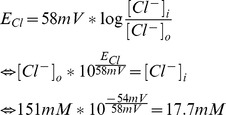



#### Calcium imaging

To measure changes of intracellular calcium levels, cells on 30 mm glass cover slips were incubated in 3 mM Fura-2/AM (Invitrogen) at 37°C and 6% CO_2_ for 60 min. Glass slides were inserted into a measuring chamber from inert steel that was mounted on an inverted microscope (Axiovert 200, Zeiss) equipped with a fluorescence-optimized 20-fold Zeiss UplanApo (20×/0.75) objective. Cells were excited intermittently for 100 ms at wavelengths of 340 nm and 380 nm (Lambda DG4, Sutter Instrument Company, connected to a Uniblitz Vmm-D1 shutter driver and a Voltakraft condensor) at 1 Hz. Emitted light with a wavelength of 510 nm was detected via a Zeiss Axiocam MRM charge-coupled-device (CCD) camera. Imaging data were acquired using the Slide-Book software (3I-Imaging, Germany). Changes of [Ca^2+^]_i_ were measured as the ratio of the 510 nm emission for both excitation wavelengths (f_340_/f_380_). At the end of every measurement, the viability and neuronal phenotype of the cells was verified by stimulation with a depolarizing buffer containing 45 mM KCl: 100 mM NaCl, 45 mM KCl, 2 mM CaCl_2_, 1 mM MgCl_2_, 10 mM HEPES. Furthermore, the buffer for Ca^2+^-free experiments contained: 140 mM NaCl, 5 mM KCl, 1 mM MgCl_2_, 5 mM EGTA, and 10 mM HEPES. Cl^−^-reduced extracellular solution contained: 140 mM NaOH, 140 mM MeSO_4_, 5 mM CsCl, 2 mM Ca^2+^ gluconate, 2 mM MgSO_4_, and 10 mM HEPES. The solutions were adjusted to pH 7.4 with NaOH/HCl and to 310 Osmol with glucose.

### Behavioral tests

NKCC1^−/−^ mice (5 ♀, 6 ♂) and WT NMRI mice (5 ♀, 5 ♂) aged 2–4 m were used for the experiments with a matched mean weight ± S.D. of 31.3 ±4.6 g (range: 25.5–38.0 g), vs. 32.3±6.3 g (range: 23.4–40.3 g), respectively. Mice were housed individually and fed normal laboratory chow *ad libitum*. Animals were subjected to 22.5 hrs of water deprivation prior to each test session. After four training days (water/water), water/water days alternated with solvent (EtOH)/capsaicin days. In a session, mice were given deionized water or water adulterated with the corresponding concentration of solvent (0.001% to 0.1% EtOH) for 30 s. After an interval of 30 s, animals were given deionized water or capsaicin-adulterated deionized water for another 30 s. The 30-s drinking interval was started after initial mouth contact with the bottle. Test solutions were presented in the home cage to minimize stress. Capsaicin concentrations (1, 3, 10, 30, 100, and 300 µM) were given in ascending order. Volume consumed was measured by an electronic scale (1 g = 1 ml). One hour after each test session, mice were given access to water for 30 min. Animal weight was controlled daily. All mice maintained their starting weight ±20% during the entire testing phase and sampled the fluid on every test.

Water consumption by NKCC1^−/−^ mice was generally lower than that by WT mice for both drinking intervals (176±9 µl vs. 242±14 µl, and 201±14 µl vs. 264±15 µl). To control for these differences, the amount of water consumed during the 2^nd^ exposure was normalized to that consumed during the 1^st^ exposure for each mouse to form an Exposure Intake Ratio.

### Data analysis and statistics

For details on NGS data analysis, see transcriptome section. Experiments on primary trigeminal ganglion neurons were performed on at least three independent preparations. Fluorescence data obtained in Ca^2+^ and Cl^−^ imaging measurements were exported as Microsoft Excel (Microsoft Corp., Seattle, USA) formatted tables from the respective imaging software. Baseline Cl^−^ and Ca^2+^ levels were determined as the mean of 10 time points prior to any stimulation. Response amplitudes were calculated using Excel macros. All dose-response curves were fitted with IgorPro (Wavemetrics, Portland, USA) using a Hill fit. Origin Pro (Systat Software Inc.) and SPSS Statistics 20 (IBM) were used for statistical analysis. Normal distribution was rejected at p≤0.05 as tested by Kolmogorov-Smirnov test. For normally distributed independent and dependent data sets, significance levels were tested by *t*-test. Non-normally distributed and/or small data sets were analyzed using the U-test (Mann-Whitney) for independent samples or the Wilcoxon rank-sum test for dependent samples. The behavioral data were analyzed for effects of genotype and capsaicin concentration on the Exposure Intake Ratio using a repeated measures ANOVA. A Bonferroni-corrected *t*-test was used to test for differences of Exposure Intake Ratio between the genotypes at the concentrations tested. All data are presented as mean ± SEM. In the figures, statistical significance is denoted as n. s. for p>0.05, * for p≤0.05, ** for p≤0.01, and *** for p≤0.001.

## Results

### Determination of the [Cl^−^]_i_ of TG neurons by intracellular calibration

**Figure 1:**
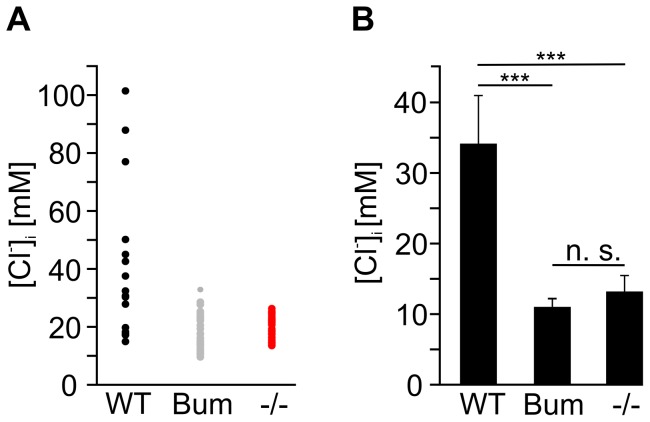
Determination of [Cl^−^]_i_ in TG neurons by MQAE-based fluorometry. A: Distribution of single cell [Cl^−^]_i_ values for TG neurons of newborn WT (n = 16), bumetanide-treated WT (n = 29), and NKCC1^−/−^ (n = 33) mice determined by intracellular calibration with defined Cl^−^ concentrations. B: Average [Cl^−^]_i_ of WT (34.1±6.9 mM, n = 16), bumetanide-treated (10.9±1.2 mM, n = 29), and NKCC1^−/−^ (13.2±1.1 mM, n = 33) mouse TG neurons. *** indicates significance at p≤0.001.

In order to determine their [Cl^−^]_i_, TG neurons isolated from newborn WT mice were loaded with the Cl^−^-sensitive dye MQAE. Intracellular Cl^−^ calibration was performed using the double-ionophore technique (see Methods section). We found an approximately Gaussian distribution of [Cl^−^]_i_ levels with single cell values ranging from 7.8 mM to 102.1 mM (n = 16). The average [Cl^−^]_i_ in WT TG neurons was 34.1±6.9 mM. The cotransporter NKCC1 is involved in intracellular Cl^−^ accumulation in different types of neurons. In order to investigate the contribution of NKCC1 to Cl^−^ accumulation in TG neurons, we pretreated the cells with the NKCC1 blocker bumetanide (50 µM) [56]. In treated cells, we determined an average [Cl^−^]_i_ of 10.9±1.2 mM (range: 2.5 mM –25.8 mM, n = 29). Accordingly, the [Cl^−^]_i_ of neurons isolated from NKCC1^−/−^ mice was 13.2±1.1 mM (range: 6.5 mM –39.6 mM, n = 33; fig. 1A, B). In the bumetanide-treated, as well as the NKCC1^−/−^ mouse TG neurons, the [Cl^−^]_i_ was significantly lower in comparison to the WT (p≤0.001, and p≤0.001, respectively). These findings demonstrate that NKCC1 cotransporter function is required for intracellular Cl^−^ accumulation in TG neurons.

**Figure 2:**
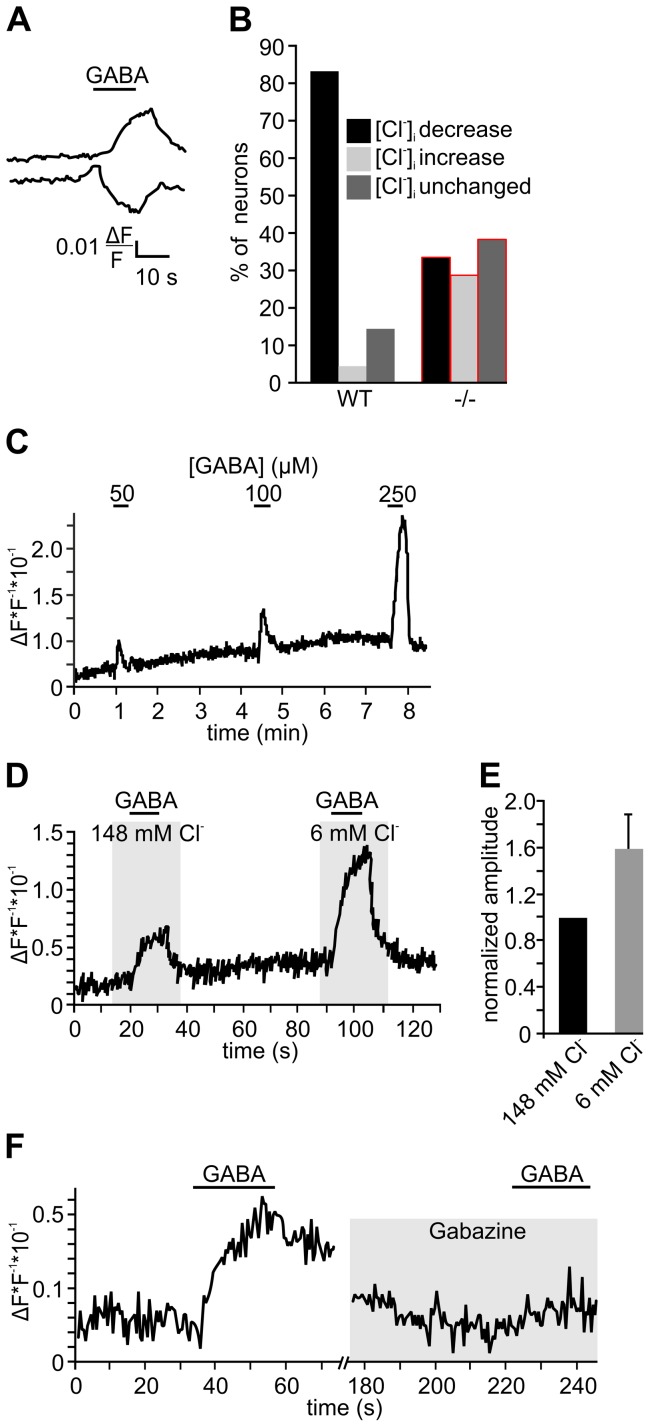
MQAE-based fluorometry of GABA-induced Cl ^−^ **responses of TG neurons.** A: Exemplary recordings of GABA-induced alterations of [Cl^−^]_i_. B: Percentage of newborn WT (n = 78) and NKCC1^−/−^ (n = 105) mouse neurons showing either a decrease, increase, or no alteration of [Cl^−^]_i_ in response to GABA (250 µM) stimulation. C: Recording of MQAE fluorescence changes of a TG neuron upon stimulation with increasing concentrations of GABA. D, E: Mean changes of MQAE fluorescence induced by 250 µM GABA in the presence of 148 mM and 6 mM Cl^−^ (n = 38). F: Inhibition of responses induced by 30 µM GABA by 10 µM of the GABA_A_ receptor antagonist gabazine (n = 25).

We next asked whether the [Cl^−^]_i_ of WT TG neurons would be high enough to give rise to Cl^−^ efflux upon the opening of Cl^−^ channels. The direction of Cl^−^ flux is determined by the difference between the Cl^−^ reversal potential (E_Cl_) and the resting membrane potential (RMP) of a cell. In mature neurons, E_Cl_ typically approximates the RMP. As a consequence, Cl^−^ currents do not tend to change the membrane potential, but stabilize the RMP and thus counteract neuronal depolarization [57]. A depolarizing Cl^−^ efflux occurs in neurons that maintain an E_Cl_ more positive than the RMP. In patch-clamp experiments on TG neurons of newborn mice, we found an average RMP of −53.9 mV±1.8 mV (−64 to −45 mV, n = 24) which is in agreement with a previous report [52]. Based on our experimental conditions (mean RMP = −54 mV, T = 22°C, [Cl^−^]_o_ = 151 mM), we calculated an E_Cl_ of −37.6 mV for WT and of −61.4 mV for NKCC1^−/−^ neurons. Furthermore, the critical [Cl^−^]_i_ for Cl^−^ efflux from TG neurons was calculated to be 17.7 mM at a RMP of −54 mV. According to our experimental data, about 70% of the TG neurons displayed a [Cl^−^]_i_ >17.7 mM and should thus show a Cl^−^ efflux upon the opening of Cl^−^-conducting ion channels. TG neurons have been demonstrated to express several GABA_A_ receptor subunits [58], [59]. We therefore suspected that GABA is a suitable stimulus to study Cl^−^ movement in TG neurons. Indeed, we could show GABA-induced responses in 100% of the newborn and adult WT, as well as newborn NKCC1^−/−^ mouse neurons in electrophysiological recordings (fig. S1). The responses were sensitive to the GABA_A_ receptor antagonist bicuculline (fig. S1). We next challenged MQAE-loaded TG neurons isolated from newborn CD1 mice with GABA (n = 78). In these life-cell experiments, we observed GABA-induced changes of [Cl^−^]_i_ within short latency after stimulus onset. Since Cl^−^ quenches MQAE fluorescence, an increase in fluorescence of MQAE represents a decrease of [Cl^−^]_i_ resulting from Cl^−^ efflux. Only fluorescence changes exceeding the baseline fluorescence more than fourfold the baseline's standard deviation were regarded as responses. Individual TG neurons displayed either an efflux, influx, or no measurable alterations of [Cl^−^]_i_ (fig. 2A). A fraction of 82.1% of NKCC1 WT littermate TG neurons displayed an decrease of [Cl^−^]_i_ in response to GABA stimulation. Furthermore, 4.1% of the neurons showed an increase and 13.8% no change of the [Cl^−^]_i_. In comparison to that, the proportions of NKCC1^−/−^ TG neurons showing an increase, a decrease, or no change of [Cl^−^]_i_ were 33.3%, 28.6%, and 38.1%, respectively (fig. 2B). Similarly, we observed GABA-induced Cl^−^ efflux in 61.5% of n = 39 adult mouse neurons (data not shown). In WT neurons, the amplitude of [Cl^−^]_i_ reduction was dependent on the GABA dose (fig. 2C). Enhancing the transmembrane driving force for Cl^−^ by reducing the [Cl^−^]_o_ led to an elevated mean MQAE fluorescence by 48.0±3% in comparison to standard conditions upon GABA application (n = 38, fig. 2D). The GABA_A_ receptor antagonist gabazine (10 µM) completely abolished the 30 µM GABA-induced increase in MQAE fluorescence (n = 25, fig. 2E).

Taken together, GABA stimulation induces a reduction of [Cl^−^]_i_ in the majority of TG neurons, most likely via Cl^−^ efflux through GABA_A_ receptor channels. In comparison to the WT, a smaller proportion of NKCC1^−/−^ neurons displayed a GABA-induced Cl^−^ efflux, but a considerable proportion rather showed Cl^−^ influx. This can be attributed to a lower [Cl^−^]_i_ in these cells that sets the E_Cl_ to values more negative than the RMP.

### GABA induces Ca^2+^ responses in TG neurons

**Figure 3:**
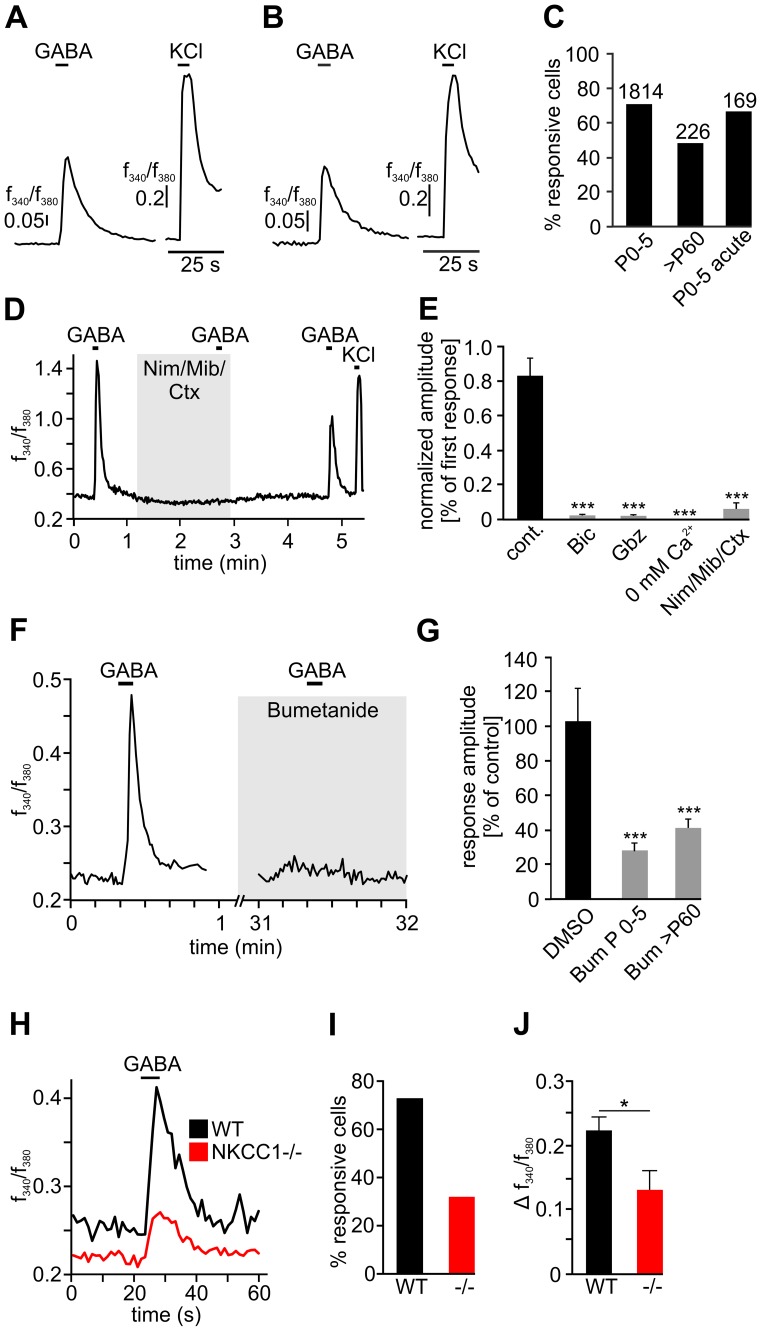
Fura2-based fluorometry of GABA-induced Ca^2+^ responses of TG neurons. A: Exemplary GABA-induced Ca^2+^ response of a newborn WT mouse neuron. B: Exemplary GABA-induced Ca^2+^ response of an adult WT mouse neuron. C: Percentage of newborn (n = 1814), adult (n = 226), and acutely dissociated newborn (n = 169) WT mouse neurons displaying 250 µM GABA-induced Ca^2+^ responses. D: Exemplary Ca^2+^ response of a newborn WT mouse TG neuron to 250 µM GABA in the presence of nimodipine (10 µM), mibefradil (10 µM), and ω-conotoxin (1 µM) (Nim/Mib/Ctx). E: Comparison of mean amplitudes of Ca^2+^ responses of WT TG neurons stimulated with 250 µM GABA under different experimental conditions. Bic  = 100 µM bicuculline (n = 53), Gbz  = 100 µM gabazine (n = 84). F: Exemplary Ca^2+^ response of a newborn mouse TG neuron to 100 µM GABA prior to and after 30 min preincubation with 50 µM bumetanide. G: Effects of 30 min preincubation with 50 µM bumetanide on GABA-induced Ca^2+^ responses of newborn (P0-5, n = 76) and adult (>P60, n = 16) WT mouse TG neurons. H: Exemplary Ca^2+^ responses of NKCC1^−/−^ and WT littermate TG neurons to GABA stimulation. I: Percentage of NKCC1^−/−^ (n = 60) and WT littermate (n = 109) TG neurons responsive to the stimulation with a saturating concentration of GABA (250 µM). J: Amplitudes of GABA-induced Ca^2+^ responses of NKCC1^−/−^ (n = 60) and WT littermate (n = 109) TG neurons. * indicates significance at p≤0.05, and *** at p≤0.001.

We next investigated whether the GABA-induced Cl^−^ efflux would be sufficient to trigger voltage-gated Ca^2+^ channel (VGCC) activation in TG neurons. Neuronal depolarization that reaches suprathreshold potential for the gating of VGCCs will lead to responses visible in Ca^2+^ imaging. Challenging fura-2/AM-loaded cells with GABA (250 µM), we found Ca^2+^ responses in a large population of newborn as well as adult mouse TG neurons (71.2%, n = 1814, and 48.7%, n = 226, respectively, fig. 3A–C). In accordance with our voltage-clamp recordings and Cl^−^ imaging experiments, GABA-induced Ca^2+^ transients were blocked by the GABA_A_ receptor antagonists bicuculline (1.5±1.5% of controls, n = 53, p≤0.001), and gabazine (1.5%±0.6% of controls, n = 84, p≤0.001). The responses were completely abolished in EGTA-buffered Ca^2+^–free extracellular solution (n = 94) and diminished to 14±0.8% (n = 175, p≤0.001) of controls in the presence of a combination of the VGCC blockers mibefradil (10 µM), nimodipine (10 µM) and ω-conotoxin MVIIC (1 µM, fig. 3D, E). Thus, the GABA-induced Ca^2+^ signals seen in TG neurons depend on extracellular Ca^2+^ that enters through VGCCs. These observations suggest that a large fraction of TG neurons is depolarized upon GABA_A_ receptor activation and that this depolarization triggers Ca^2+^ influx through VGCCs. The prerequisite for GABA-induced depolarization is an efflux of Cl^−^ along its electrochemical gradient which in turn requires intracellular Cl^−^ accumulation. To test whether NKCC1 transporter activity sustained the GABA-induced Ca^2+^ transients seen in TG neurons, we treated newborn and adult mouse TG neurons with the NKCC1 inhibitor bumetanide (50 µM, 30 min) prior to GABA application. The amplitudes of GABA-induced Ca^2+^ transients were diminished to 29.6±1.4% of controls in newborn (n = 76, p≤0.001) and to 41.3±5.4% in adult mouse neurons (n = 16, p≤0.001) after bumetanide pretreatment (fig. 3F, G). Furthermore, we found responses to GABA in 31.7% of the NKCC1^−/−^ mouse TG neurons (n = 60, fig. 3H, I). In correspondence to the bumetanide-treated WT neurons, the mean response amplitudes of NKCC1^−/−^ neurons were 40% smaller in comparison to the littermate controls (n = 60 and n = 109, respectively, p≤0.01, fig. 3J), but insensitive to bumetanide treatment (not shown). Taken together, our findings strongly point to a Cl^−^ efflux-induced depolarization of TG neurons upon GABA stimulation. This depolarization induces Ca^2+^ influx via VGCCs and NKCC1 function is required for the depolarizing effect of GABA.

### Next generation sequencing and PCR reveal CaCC transcripts in trigeminal ganglia

**Figure 4:**
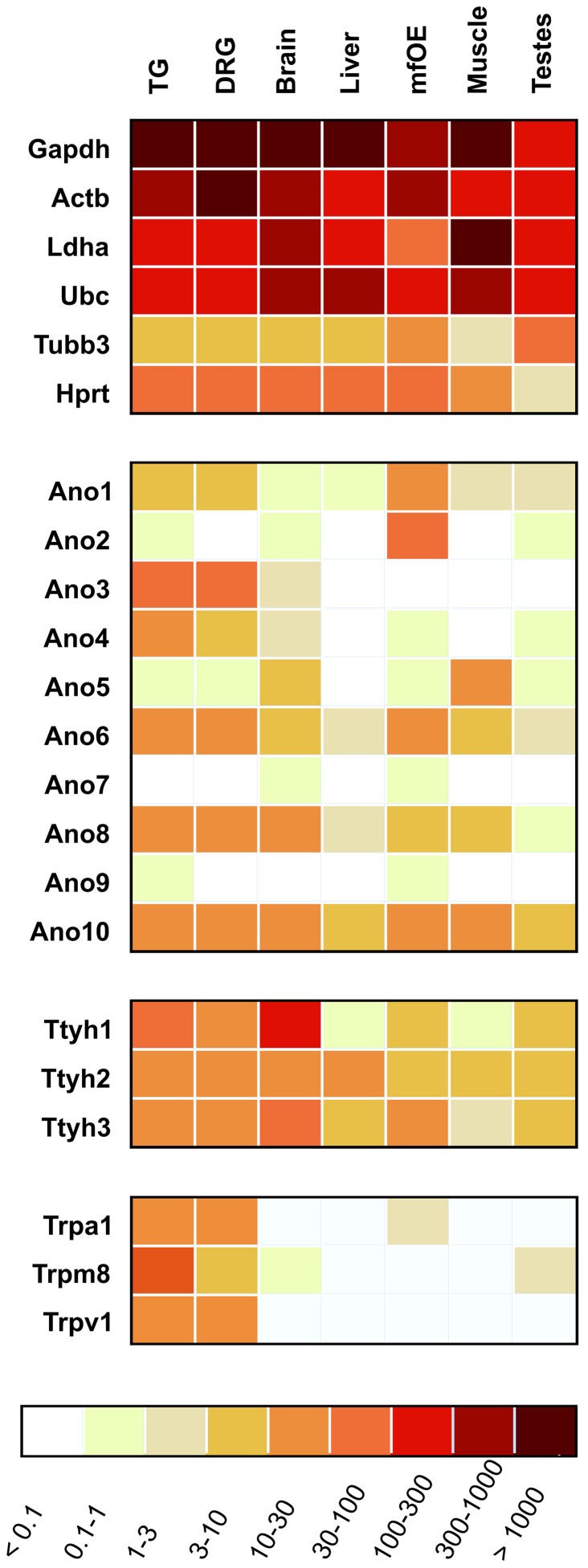
Determination of CaCC transcript levels by next generation sequencing. Heat map showing the expression levels of different CaCCs in chemosensory tissue (trigeminal ganglia (TG), dorsal root ganglia (DRG), and olfactory epithelium (OE)) of adult CD1 mice sampled by our group, and of brain, liver, muscle [47], and testis [48]. Higher FPKM values are indicated by deeper color. Gapdh: glyceraldehyde-3-phosphate dehydrogenase, Actb: actin, cytoplasmic 1, Ldha: L-lactate dehydrogenase A chain isoform 2, Ubc: polyubiquitin-C, Tubb3: tubulin beta-3 chain, Hprt: hypoxanthine-guanine phosphoribosyltransferase, Ano1-10: anoctamin1-10, Ttyh1: protein tweety homolog 1 isoform 1, Ttyth2, 3: protein tweety homolog 2, 3, TRPA1: transient receptor potential cation channel subfamily A member 1, TRPM8: transient receptor potential cation channel subfamily M member 8, TRPV1: transient receptor potential cation channel subfamily V member 1.

**Figure 5:**
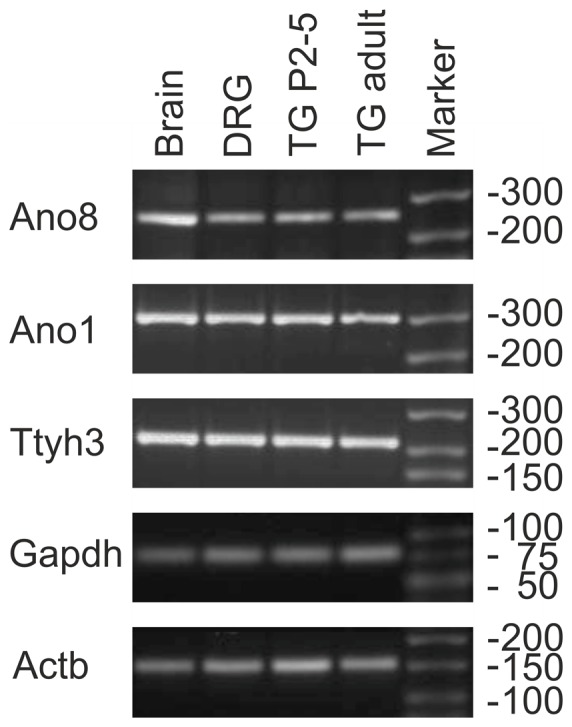
PCR for Ano1, Ano8, and tweety3 in different neuronal tissues. Representative PCR for Ano1, Ano8, and tweety3 on adult and newborn mouse TG, and adult mouse DRG and brain tissue. Ano1, 8: anoctamin1, 8, Ttyth3: protein tweety homolog 3, Gapdh: glyceraldehyde-3-phosphate dehydrogenase, Actb: actin, cytoplasmic 1.

Numerous stimuli of TG neurons activate Ca^2+^-conducting ion channels. This leads to an elevation of cytosolic Ca^2+^ which might activate CaCCs in the course of a downstream signal amplification cascade as seen in OSNs. We asked whether TG neurons are equipped for generating Ca^2+^-activated Cl^−^ currents. To identify transcripts of candidate CaCCs, we analyzed transcriptome data generated by RNA-Seq of TG, DRG, and OE tissue from adult CD1 mice and calculated FPKM values for different gene families coding for CaCCs, neuronal markers, as well as housekeeping genes (table S1). In both, complete DRG and TG, we could show the expression of several CaCCs of the Anoctamin and Tweety gene families (fig. 4). Comparing all tissues tested, the expression levels of CaCCs were highest in TG and DRG as well as OE. Expression level in terms of FPKM values can be compared to housekeeping genes and allow a rough classification as low (FPKM ∼1), medium (∼10), or highly expressed (∼100) genes (table S1). We found high expression levels of members of the Anoctamin (TMEM16) family as well as tweety transcripts in TG tissue (fig. 4). The transcript level of Ano2 (aka TMEM16B), the CaCC involved in olfactory sensing was very low in the TG compared to the OE (FPKM values: 0.4 vs. 58.3). Along with Ano1, it has not been detected by immunohistochemistry in nasal trigeminal sensory fibers in a previous study [60]. Ano1 is a channel involved in the bradykinin-mediated depolarization and heat response of DRG neurons [43], [44] and thus appears to be of importance in peripheral sensory neurons. In our samples, it was expressed at comparably low levels with an FPKM value of 4.5. Higher levels of Ano1 mRNA were identified in OE tissue (FPKM value of 12.2). We found comparably high FPKM values for Ano3, Ano4, and Ano6 mRNA (48.3, 9.4, and 17.8, respectively). However, the proteins were described to be located intracellularly [61] and, thus, most likely are not involved in mediating the signaling events we observed. Ano8 and Ano10 give rise to transmembrane Ca^2+^-activated Cl^−^ currents [62]. In the TG, we found FPKM values of 12.1 and 15.7 for these channels, respectively (OE: 6.0 and 8.3, brain: 16.7 and 12.9). However, Ano10 is a slowly responding channel [62] and, thus, most likely not involved in the fast signaling events we observed. As another interesting CaCC, we found high levels of tweety3 mRNA that codes for a plasma membrane channel conducting macroscopic Cl^−^ currents [39], [63]. FPKM values for the different CaCCs in chemosensory tissues sequenced by our group or resulting from our analysis of published raw RNA-seq data for brain, liver, muscle [47], and testis [48] are presented as a heat map in fig. 4. For comparison, the FPKM values for the three most prominent chemosensory TRP channels in DRG and TG, namely TRPV1, TRPM8, and TRPV1, are also given. We were able to confirm the expression of the most interesting CaCCs Ano1, Ano8, and tweety3 in the TG and DRG by PCR. Transcripts were verified in tissue samples of adult as well as newborn mouse TG, adult mouse DRG, and adult mouse brain (fig. 5, see figure S2 for control PCR).

In summary, screening the adult TG for CaCC transcripts, we found expression of different CaCCs with Ano1, Ano8, and tweety3 being the most interesting candidate channels. Thus, we assume that the TG is equipped for Cl^−^-dependent signal amplification via Ca^2+^-triggered Cl^−^ efflux.

### TG neurons exhibit CaCC functionality

**Figure 6:**
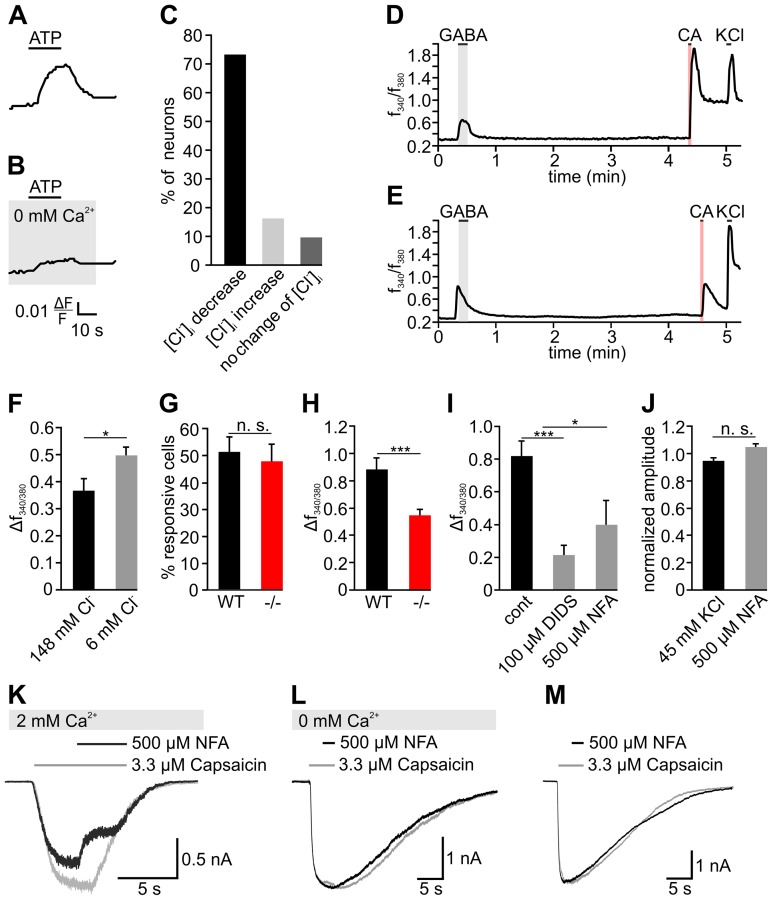
Functional characterization of Ca^2+^-induced Cl^−^ responses of TG neurons. A–C show results of Cl^−^ imaging experiments. With the exception of E, all experiments were performed on newborn mouse neurons. A, B: 100 µM ATP-stimulated Cl^−^ efflux in TG neurons in the presence and absence of extracellular Ca^2+^ (n = 18). C: Quantification of neurons displaying either a decrease, an increase, or no alteration of [Cl^−^]_i_ upon ATP stimulation (n = 76). D–J show results of Ca^2+^ imaging experiments. Representative Ca^2+^ responses of a newborn (D, n = 1178) and adult mouse TG neuron (E, n = 169) to stimulation with saturating concentrations of GABA (250 µM) and capsaicin (3.3 µM). F: Mean Ca^2+^ response amplitudes to 3.3 µM capsaicin in saline containing 140 mM or 6 mM [Cl^−^] (n = 42). G: Percentage of WT (n = 62) and NKCC1^−/−^ (n = 101) TG neurons responsive to capsaicin. H: Mean Ca^2+^ response amplitudes to 3.3 µM capsaicin in WT (n = 62) and NKCC1^−/−^ (n = 101) TG neurons. I: Effects of the CaCC blockers DIDS (100 µM) and NFA (500 µM) on the mean amplitudes of 3.3 µM capsaicin-induced Ca^2+^ responses in WT TG neurons. J: Effects of NFA treatment on depolarization-induced Ca^2+^ responses of TG neurons (n = 94). K–M: Patch clamp characterization of capsaicin-induced responses. K: Exemplary response of a TG neuron to capsaicin in the presence and absence of 500 µM NFA (n = 9, p≤0.01). L: Effect of NFA on the capsaicin-induced current of a TG neuron in the absence of extracellular Ca^2+^ (n = 12, n. s.). M: Effect of NFA on the capsaicin-induced current of a HEK/mTRPV1 cell (n = 10, n. s.). * indicates significance at p≤0.05, and *** at p≤0.001.

After we identified high levels of different CaCC transcripts in the TG, we tested whether intracellular Ca^2+^ elevations could induce alterations of Cl^−^ levels in TG neurons. Unfortunately, the prototypic trigeminal agonist capsaicin shows auto fluorescence in Cl^−^ imaging experiments (not shown). Thus, we chose ATP as a stimulus that elicits Ca^2+^ signals in at least 90% of rodent TG neurons [64]. Here, ATP activates cation-permeable P2X ion channels [64]. Beyond that, immunohistochemical as well as functional analysis revealed the presence of phospholipase C (PLC)-coupled P2Y receptors in TG neurons [65], [66]. In Cl^−^ imaging experiments, ATP (100 µM) challenge induced a Cl^−^ efflux in newborn mouse TG neurons (fig. 6A). In more detail, we observed a decrease in 74.2%, an increase in 16.1%, and no change of [Cl^−^]_i_ in 9.7% of all TG neurons tested (n = 76, fig. 6C) which is reminiscent of the results we observed upon GABA application. In the absence of extracellular Ca^2+^, ATP stimulation induced only minor changes in MQAE fluorescence (n = 18, fig. 6B). Apparently, the ATP-induced elevation of cytosolic Ca^2+^ resulting mainly from P2X receptor activation leading to entry of extracellular Ca^2+^, but not of P2Y receptor activation that induces Ca^2+^ release from the endoplasmic reticulum, is required for alterations of [Cl^−^]_i_.

Next, we investigated the possible involvement of CaCCs in a Cl^−^-dependent signal amplification mechanism in TG neurons. Therefore, we tested whether capsaicin-sensitive TG neurons would also display GABA-induced Ca^2+^ transients in Ca^2+^ imaging experiments. Capsaicin sensitivity was present in 45.6% (537/1178) of the newborn and 40.8% (69/169) of the adult mouse neurons. Of newborn and adult mouse capsaicin-sensitive neurons, 68.8% and 63.8% also displayed GABA-induced Ca^2+^ transients (fig. 6D, E). This finding argues for augmented Cl^−^ levels in about two thirds of all trigeminal capsaicin sensors, irrespective of animal age. Given the expression of different CaCCs in the TG and the ATP-induced and Ca^2+^-dependent Cl^−^ efflux seen in most TG neurons, we suspected that capsaicin-induced Ca^2+^ influx might trigger a CaCC-dependent Cl^−^ efflux. This in turn might elevate the overall activation of TG neurons, possibly via VGCCs. Thus, an enhancement of the outward driving force for Cl^−^ should increase the amplitudes of capsaicin responses. To test this, we applied capsaicin in extracellular buffer with reduced [Cl^−^]_o_. Under these conditions, the mean amplitude of capsaicin-induced Ca^2+^ transients was increased by 35% (n = 42, p≤0.05, fig. 6F). Conversely, a lower [Cl^−^]_i_ should reduce the outward driving force for Cl^−^ and consequently lead to smaller Ca^2+^ transients resulting from a less pronounced depolarization upon capsaicin stimulation. Indeed, the mean amplitudes of capsaicin-induced Ca^2+^ transients were reduced by 38.5% in NKCC1^−/−^ mouse TG neurons compared to the WT (n = 62 and n = 101, p≤0.001 respectively, fig. 6H). In comparison to controls, a similar fraction of NKCC1^−/−^ mouse neurons was sensitive to capsaicin stimulation (51.2±5.6% vs. 47.7±6.1%) showing that NKCC1 knockout did not generally impair the capsaicin sensitivity of TG neurons (fig. 6G). We next applied capsaicin together with the CaCC inhibitors DIDS or NFA to newborn mouse neurons. In the presence of 100 µM DIDS, the mean amplitudes of capsaicin-induced Ca^2+^ transients were reduced to 73.7% of controls (n = 14, p≤0.001). Similarly, we found a reduction to 51.3% of controls in the presence of 500 µM NFA (n = 13, p≤0.05, fig. 6I). At this concentration, NFA did not diminish Ca^2+^ transients evoked by depolarization with buffer containing 45 mM KCl (n = 94, n. s., fig. 6J). We can therefore exclude an inhibition of VGCCs by NFA. In patch-clamp experiments, capsaicin (3.3 µM) evoked robust inward currents that were reduced by 37.5±2.9% (n = 9) in the presence of 500 µM NFA (n = 9, p≤0.01, fig. 6K). NFA did not exert any effect on capsaicin-induced currents in TG neurons under Ca^2+^-free conditions (n = 12, n. s., fig. 6L). Moreover, NFA did not inhibit capsaicin-induced responses of mTRPV1-expressing HEK293 cells excluding unspecific effects of the drug on the receptor (n = 10, n. s., fig. 6M).

Taken together, our data show the amplification of capsaicin-induced responses in a subpopulation of capsaicin-sensitive TG neurons that depends on the transmembrane driving force for Cl^−^ and that is sensitive to inhibitors of CaCCs.

### NKCC1^−/−^ mice show a higher tolerance for oral capsaicin

**Figure 7:**
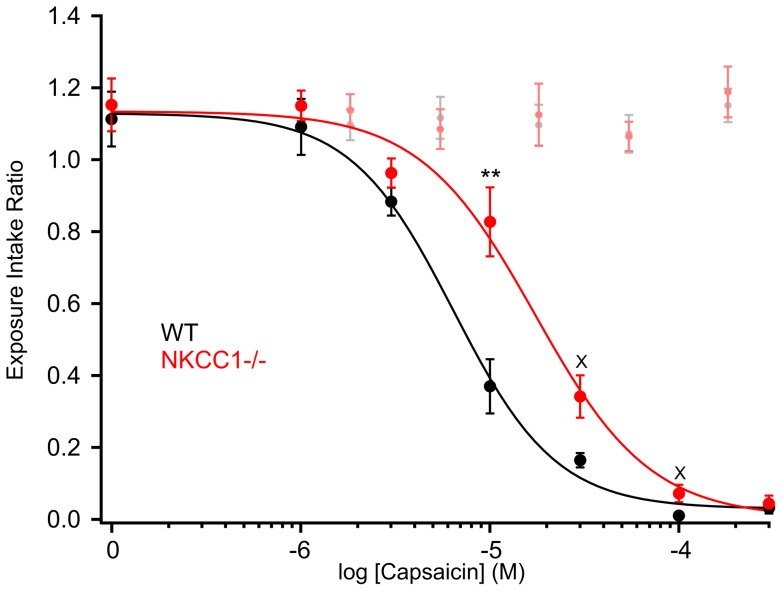
Mean Exposure Intake Ratios (± SEM) for various capsaicin concentrations presented on the 2^nd^ 30-s fluid exposure following a 30-s solvent exposure and a 30-s rest for adult WT (n = 10) and NKCC1^−/−^ (n = 11) mice. The EC_50_ of capsaicin in water was 6.4±0.7 µM for the WT and 17.3±3.2 µM for the NKCC1^−/−^ mice. The NKCC1^−/−^ had capsaicin concentration-avoidance functions that were shifted to the right by 0.37 log_10_ units. Pale points represent performance on control sessions in which water was presented on both trials. Asterisks represent statistically significant difference (**p≤0.01) between the genotypes as revealed by post hoc *t*-test. X indicates significant difference (p≤0.05) that did not survive Bonferroni correction.

In Ca^2+^ imaging experiments, we found GABA-induced Ca^2+^ transients indicating GABA-mediated depolarization in about two thirds of capsaicin-sensitive adult mouse TG neurons. Furthermore, NKCC1^−/−^ TG neurons exhibited smaller Ca^2+^ response amplitudes upon capsaicin stimulation. Thus, we reasoned that the disturbed Cl^−^ accumulation in TG neurons could reduce the sensitivity of NKCC1^−/−^ mice for capsaicin. To this end, we compared 10 WT and 11 age- and gender-matched NKCC1^−/−^ mice for their consumption of capsaicin-adulterated water. We hypothesized that the animals would avoid the capsaicin-adulterated water proportional to the intensity of the normally repellent stimulus. In each test trial, individual thirsty mice had short access to water or solvent (1^st^ exposure) followed by a 30-s pause and then again had short access to a bottle containing either water or water adulterated with capsaicin (2^nd^ exposure). The Exposure Intake Ratios calculated for the water/water trials did not differ between the NKCC1^−/−^ and WT mice (1.15±0.07 and 1.11±0.07, respectively, p>0.7). A repeated measures ANOVA (genotype x capsaicin concentration) of Exposure Intake Ratios revealed a significant main effect of genotype (F(1,19)  = 16.82, p≤0.001) and capsaicin concentration (F(3,56) = 155.79, p≤0.001, Greenhouse-Geisser correction) as well as a significant interaction (F(3,56) = 3.98, p≤0.05, Greenhouse-Geisser correction). In the solvent/capsaicin trials, NKCC1^−/−^ and WT mice almost completely refused water containing 300 µM capsaicin. However, NKCC1^−/−^ mice had Exposure Intake Ratios twice as large for either 10 µM or 30 µM capsaicin compared to the WT (0.83±0.1 vs. 0.37±0.08 and 0.34±0.06 vs. 0.17±0.02, respectively) and seven times as large at a concentration of 100 µM capsaicin (0.07±0.02 vs. 0.01±0.01). The EC_50_ of the mean capsaicin concentration-avoidance curve was 6.4±0.7 µM for the WT and 17.3±3.2 µM for the NKCC1^−/−^ mice. Accordingly, the NKCC1^−/−^ had capsaicin concentration-avoidance functions that were shifted to the right by 0.37 log_10_ units. These observations suggest a higher tolerance of NKCC1^−/−^ mice towards the aversive stimulus capsaicin (fig. 7). The behavioral difference seen for capsaicin avoidance is in line with the reduced Ca^2+^ responses of NKCC1^−/−^ mouse TG neurons in Ca^2+^ imaging.

Taking together our observations, we suggest that the reduced avoidance of NKCC1^−/−^ mice for capsaicin can be attributed to a less efficient Cl^−^-dependent signal amplification mechanism in these animals' TG neurons due to the lack of intracellular Cl^−^ accumulation by NKCC1 leading to a weaker capsaicin-evoked sensory signal.

## Discussion

Cl^−^ plays a pivotal role in basic neuronal functions like excitability, signal amplification, and signal transmission. Intracellular Cl^−^ levels can be dynamically regulated to adjust the sensitivity of individual neurons and entire neuronal networks. In this study, we describe the Cl^−^ homeostasis of trigeminal sensory neurons and a role for Cl^−^ as a signal amplifier in trigeminal sensing.

Using MQAE-based fluorometry, we identified a mean [Cl^−^]_i_ of 34 mM in isolated newborn mouse TG neurons which is somewhat lower than that described for DRG neurons of newborn mice (77 mM) [67], or rats (44 mM) [6]. However, the inter-cell variability of [Cl^−^]_i_ we found in TG neurons is comparable to that of DRG neurons. According to the typical RMP of TG neurons and the given experimental conditions, we calculated a critical [Cl^−^]_i_ of ≥17.7 mM that should give rise to GABA-induced chloride efflux. Using patch-clamp, we found GABA sensitivity in all TG neurons. Thus, 70% of all TG neurons should display GABA-induced Cl^−^ efflux according to the [Cl^−^]_i_ of individual neurons. However, in the actual stimulation experiment, more than 83% of the neurons displayed GABA-induced Cl^−^ efflux. Thus, we slightly underestimated the [Cl^−^]_i_. In Ca^2+^ imaging experiments, GABA stimulation induced Ca^2+^ responses in 71% of all TG neurons. As these responses were sensitive to blockers of VGCCs, we conclude that they depend on a depolarizing Cl^−^ efflux. In a small percentage of neurons, the Cl^−^ efflux apparently was insufficient to activate VGCCs, explaining the discrepancy between the percentages of neurons showing a GABA-induced Cl^−^ efflux and those showing Ca^2+^ transients. From the Nernst equation, we calculated an average E_Cl_ of −37.6 mV for WT and of −61.4 mV for NKCC1^−/−^ TG neurons. Thus, the opening of Cl^−^ channels will lead to a depolarizing Cl^−^ efflux in most WT neurons, but Cl^−^ influx will hyperpolarize most NKCC1^−/−^ neurons.

Unfortunately, we could not determine the [Cl^−^]_i_ of adult mouse TG neurons because of recurring membrane rupture during intracellular calibration with ionophores. Nevertheless, GABA-induced Cl^−^ efflux could be observed in over 60% of adult mouse TG neurons and close to 50% showed GABA-induced Ca^2+^ responses that were sensitive to bumetanide. We therefore conclude that at least half of the adult TG neurons generate an E_Cl_ more positive than the RMP as a result of NKCC1 activity.

In DRG neurons and OSNs, the 12TM cotransporter NKCC1 generates high levels of intracellular Cl^−^ [22], [24], [25], [67]. In the TG, the expression of NKCC1 mRNA has been demonstrated [68], [69]. In our study, pharmacological inhibition of NKCC1 resulted in a lower average [Cl^−^]_i_ and fewer neurons showing Cl^−^ efflux or Ca^2+^ transients when challenged with GABA. Similar results were obtained from NKCC1^−/−^ mouse neurons. Thus, we suggest that NKCC1 is mainly if not solely responsible for intracellular Cl^−^ accumulation in TG neurons.

In OSNs, a relatively high [Cl^−^]_i_ underlies the amplification of odor-induced signals via Cl^−^ efflux [23], [28] mediated by the CaCC TMEM16B (Ano2) [31], [32]. Ca^2+^-activated currents have also been described in neurons of the rat DRG and quail TG [40], [41], [70], [71]. In patch-clamp experiments, 50% of rat DRG neurons displayed Ca^2+^-activated Cl^−^ currents that are thought to mediate after depolarization following action potentials [72]. Interestingly, we observed a Cl^−^ efflux in TG neurons upon ATP challenge, a stimulus known to induce cytoplasmic Ca^2+^ signals in more than 90% of rodent TG neurons [64]. Assuming that all ATP-induced changes of [Cl^−^]_i_ (increase as well as decrease) are mediated by CaCCs, the percentage of CaCC-expressing TG neurons approximates 90%. Furthermore, Ca^2+^ signals induced by the prototypic trigeminal stimulus capsaicin were smaller in bumetanide-treated TG neurons and NKCC1^−/−^ TG neurons, thus, in neurons with a decreased outward driving force for Cl^−^. Taken together, we propose that Ca^2+^ signals in TG neurons trigger a depolarizing Cl^−^ efflux along its electrochemical gradient. Similar to OSNs, this Cl^−^ efflux appears to contribute to signal amplification in TG neurons. Yet, to what extent will TG neurons be depolarized by Cl^−^ efflux? Cl^−^ efflux will drive the membrane potential towards E_Cl_ (about −30 mV in WT TG neurons). Capsaicin activation in turn drives the membrane potential towards E_TRPV1_ which is about 0 mV, typical for an unspecific cation conductance. However, in a neuron that generates capsaicin-induced Cl^−^ efflux via CaCCs, the membrane potential may be stabilized at E_Cl_. This would counteract a depolarization of the neuron towards E_TRPV1_, but increase the net influx of Ca^2+^ and Na^+^ via TRPV1 according to the stabilized driving force for both cations.

Which could be the effectors of a presumed Ca^2+^-induced Cl^−^ efflux in TG neurons? Analysis of adult TG tissue by NGS revealed high levels of different CaCC transcripts. We found weak expression of the “olfactory CaCC” TMEM16B, confirming existing data [60]. However, in contrast to the very same study, we found Ano1 expression in TG tissue, albeit at comparably low levels. This may be attributable to the fact that we analyzed complete TG containing not only neurons but also other cell types, foremost satellite glia, whereas Dauner and coworkers studied nasal trigeminal fibers. In DRG neurons, Ano1 is involved in the bradykinin-mediated depolarization [43] and heat sensitivity [44]. Thus, it is likely to be of relevance in TG neurons as well. As another candidate channel, we found high levels of Ano8 mRNA. Ano8 is a plasma membrane CaCC and thus apt to mediate a Ca^2+^-activated Cl^−^ current involved in fast signaling events. We furthermore found Ano3, Ano4, Ano5, Ano6, andAno7 mRNAs at different expression levels. However, they code for intracellularly located proteins [61] and thus most likely are not involved in signal amplification in TG neurons. Ano10 most likely is not involved in fast signaling as it displays slow activation kinetics in the range of minutes [62]. A further candidate CaCC in TG neurons is Tweety3 of which we also found high mRNA levels. Tweety3 has been characterized as a plasma membrane channel conducting macroscopic Ca^2+^-activated Cl^−^ currents [39], [63]. Besides sensory neurons, other cell types are found within the TG with satellite glia being the most abundant. Thus, our NGS and PCR data show the expression of CaCCs in neuronal and non-neuronal cells. Future studies should address the detailed localization of the candidate CaCCs we present here in the TG. In addition to the identification of CaCC transcripts by means of transcriptome analysis and PCR, we could show the functionality of CaCCs in TG neurons. When increasing the driving force for a depolarizing Cl^−^ efflux capsaicin-induced Ca^2+^ responses were increased. Beyond that, the response amplitudes of NKCC1^−/−^ TG neurons stimulated with capsaicin were dramatically lower than that of the WT. Furthermore, the responses to capsaicin were sensitive to the CaCC blockers NFA and DIDS. To our knowledge, we are the first to demonstrate that Cl^−^ mediates a significant proportion of the capsaicin-induced response of somatosensory neurons. Interestingly, in our pharmacological experiments DIDS showed higher blocker efficiency towards capsaicin-induced responses than NFA. I_Cl(Ca)_ carried by Tweety3 has been described to be insensitive to NFA but was completely blocked by 10 µM DIDS [39]. Ano channels, however, are sensitive for both substances. Thus, judging from the pharmacological experiments, we assume that at least one Ano type CaCC together with Tweety3 is functional in TG neurons. According to this hypothesis, NFA will inhibit only Ano type CaCCs (lower blocker efficacy) whereas DIDS will inhibit Ano type CaCCs together with Tweety3 (high efficacy). Based on our *in vitro* findings, we hypothesized that NKCC1^−/−^ mice might be less sensitive for oral capsaicin, a stimulus of polymodal Aδ- and nociceptive C-fibers [73]. Indeed, in the short-term drinking procedure knockout animals displayed significantly less avoidance of capsaicin solutions compared to the WT. In accordance with that, NKCC1^−/−^ mice display longer withdrawal latencies in the hot plate test [21]. In concert with a role of NKCC1 in promoting the perception of painful stimuli, the pharmacological block of the transporter inhibited different nociceptive modalities, such as itch, injury-induced pain, and dorsal root reflexes in previous studies [15]–[17].

Collectively considering our *in vitro* and *in vivo* findings, what could be the reasons for the higher capsaicin tolerance by NKCC1^−/−^ mice? One possible explanation involves the regulation of signal transmission between the first and second order neuron of the trigeminal sensory system. Information transmitted by sensory neurons of the peripheral nervous system undergoes signal modulation at the first central synapse. The underlying process, primary afferent depolarization (PAD), involves the GABA-mediated depolarization of GABA_A_ receptor-bearing afferent synapses that maintain a high [Cl^−^]_i_ through NKCC1-mediated Cl^−^ accumulation. Depending on the [Cl^−^]_i_ of the afferent, the outcome of PAD can either be a signal depression or amplification. Under normal conditions, GABA release from interneurons onto the afferent synapse induces a depolarizing efflux of Cl^−^ along its electrochemical gradient that inactivates voltage-gated channels and/or shunts incoming excitation. As a result transmitter release by the afferent synapse is reduced. Pathological conditions can cause an increase of [Cl^−^]_i_ in DRG neurons by phosphorylation, recruitment, or upregulation of the transporter [10]–[14]. A higher [Cl^−^]_i_ is thought to shift the effect of PAD towards a stronger depolarization of the afferent synapse that, in contrast to a weak depolarization, is sufficient to trigger the concerted gating of a large fraction of voltage-gated ion channels, including VGCCs. The net effect is a very substantial depolarization of the afferent synapse leading to a stronger Ca^2+^ influx and consequently more transmitter release. Thereby, the activation of the second order neuron is enhanced (see also references [2] and [3]). In contrast to that, a lower [Cl^−^]_i_ as seen in NKCC1^−/−^ TG neurons should induce pronounced hyperpolarization of the presynapse, reducing the overall signal output of the sensory system. Apart from a shift of PAD to an inactivating effect, our study suggests another explanation for the reduced nocifensive behavior of the knockout. As a consequence of a lower [Cl^−^]_i_, capsaicin-induced Ca^2+^ influx via TRPV1 will produce only a weak if any Cl^−^ efflux through CaCCs in NKCC1^−/−^ TG neurons. Therefore, the afferent neurons of NKCC1^−/−^ mice generate weaker signals in response to capsaicin than those of the WT. It is perceivable that both, a shift of PAD towards hyperpolarization together with a reduction of Cl^−^-dependent signal amplification result in the reduced nocifensive behavior of NKCC1^−/−^ mice. Interestingly, knockout animals showed the same avoidance of water adulterated with the highest concentration of capsaicin used in the test (300 µM). We presume that at high capsaicin concentrations, a reduction of signal output by hyperpolarizing PAD as well as the lack of Cl^−^-based signal amplification will be overcome by a very robust activation of the afferent neurons even in NKCC1^−/−^ mice.

Capsaicin is a stimulus of nociceptive afferents. Here, we show the amplification of capsaicin-induced signals of TG neurons by Cl^−^ efflux and reduced nocifensive behavior in mice with compromised Cl^−^ accumulation in TG neurons. The inhibition of CaCCs was shown to inhibit bradykinin-induced pain [43]. In our view, the idea of a Ca^2+^-activated, Cl^−^-dependent component of pain perception should be followed in further studies, also with the prospect of developing new analgesic agents.

## Supporting Information

Figure S1 **Patch clamp characterization of GABA-induced currents in TG neurons.** A: Exemplary responses of TG neurons to stimulation with GABA (100 µM). Cells were kept at a holding potential of −60 mV. The total number of responsive cells was 45/45 (newborn WT), 20/20 (adult WT), and 15/15 (newborn NKCC1−/−). Bars signify duration of stimulus application. B, C: Dose-response curve for GABA at newborn WT TG neurons (n = 15) displaying an EC_50_ of 24.7±1.6 µM and a Hill coefficient of 2.1±0.3. D, E: Dose-dependent inhibition of responses induced by 30 µM GABA by the GABA_A_ receptor antagonist bicuculline with an IC_50_ of 1.3±0.2 µM (n = 12).
(TIF)

Figure S2 **Control PCR for Ano1, Ano8, and tweety3 in different neuronal tissues.** For the minus-RT PCR, RNA prior to cDNA synthesis was used as template. PCR was performed under the same condition as for the cDNA analysis. 1: adult female TG, 2: adult brain, 3: adult DRG, 4: newborn mouse (P2-5) TG, and 5: adult male TG.
(TIF)

Table S1 **FPKM values for different housekeeping, Ca^2+^-gated Cl^−^ channel, and TRP channel genes in murine neuronal and non-neuronal tissues.**
(DOCX)
