# Supplementary figures and images for: Trigeminal Ganglion Neurons of Mice Show Intracellular Chloride Accumulation and Chloride-Dependent Amplification of Capsaicin-Induced Responses
Source: PLoS One. 2012 Nov 8;7(11):e48005. doi: 10.1371/journal.pone.0048005 (PMC3493563; doi:10.1371/journal.pone.0048005)

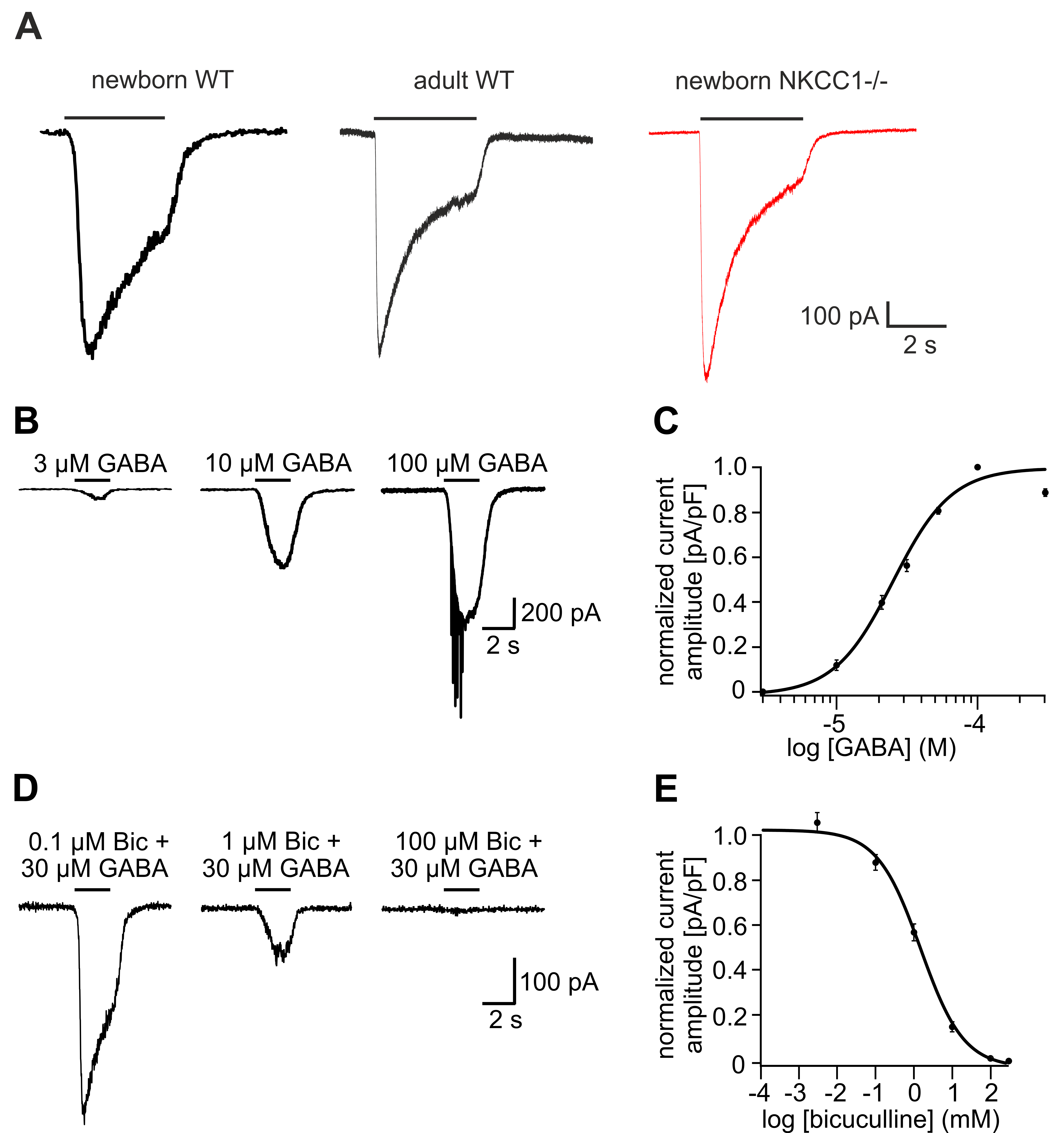

Supplement: Figure S1 — Patch clamp characterization of GABA-induced currents in TG neurons. A: Exemplary responses of TG neurons to stimulation with GABA (100 µM). Cells were kept at a holding potential of −60 mV. The total number of responsive cells was 45/45 (newborn WT), 20/20 (adult WT), and 15/15 (newborn NKCC1−/−). Bars signify duration of stimulus application. B, C: Dose-response curve for GABA at newborn WT TG neurons (n = 15) displaying an EC50 of 24.7±1.6 µM and a Hill coefficient of 2.1±0.3. D, E: Dose-dependent inhibition of responses induced by 30 µM GABA by the GABAA receptor antagonist bicuculline with an IC50 of 1.3±0.2 µM (n = 12). (TIF) [file pone.0048005.s001.tif]

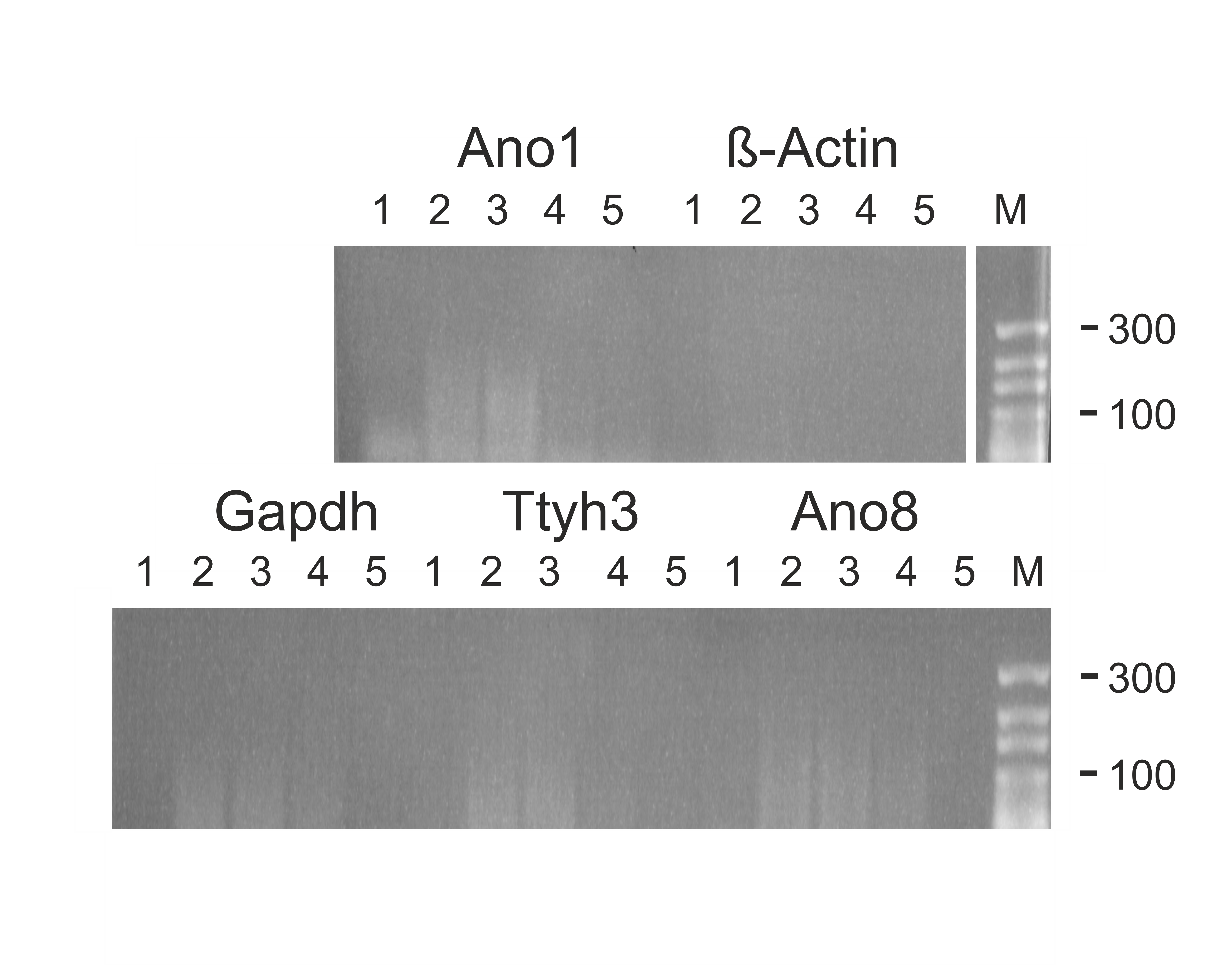

Supplement: Figure S2 — Control PCR for Ano1, Ano8, and tweety3 in different neuronal tissues. For the minus-RT PCR, RNA prior to cDNA synthesis was used as template. PCR was performed under the same condition as for the cDNA analysis. 1: adult female TG, 2: adult brain, 3: adult DRG, 4: newborn mouse (P2-5) TG, and 5: adult male TG. (TIF) [file pone.0048005.s002.tif]
