# Supplementary material for: Trigeminal Ganglion Neurons of Mice Show Intracellular Chloride Accumulation and Chloride-Dependent Amplification of Capsaicin-Induced Responses
Source: PLoS One. 2012 Nov 8;7(11):e48005. doi: 10.1371/journal.pone.0048005 (PMC3493563; doi:10.1371/journal.pone.0048005)
Supplement: Table S1 — FPKM values for different housekeeping, Ca2+-gated Cl− channel, and TRP channel genes in murine neuronal and non-neuronal tissues. (DOCX) [file pone.0048005.s003.docx]

Supplementary table 1: FPKM values for different housekeeping, Ca^2+^-gated Cl^-^ channel, and TRP channel genes in murine neuronal and non-neuronal tissues.

| Gene ID | Brain | Liver | mfOE | Muscle | Testes | TG | DRG | Description |
| --- | --- | --- | --- | --- | --- | --- | --- | --- |
| Actb | 391.0 | 225.1 | 922.0 | 191.8 | 177.6 | 926.3 | 1093.6 | actin, cytoplasmic 1 |
| Ano1 | 0.8 | 0.8 | 19.4 | 1.7 | 2.4 | 5.3 | 3.2 | anoctamin-1 isoform 1 |
| Ano2 | 0.4 | 0.0 | 102.6 | 0.0 | 0.3 | 0.5 | 0.0 | anoctamin-2 |
| Ano3 | 2.7 | 0.0 | 0.0 | 0.0 | 0.0 | 52.2 | 52.2 | anoctamin-3 isoform 1 |
| Ano4 | 2.1 | 0.0 | 0.3 | 0.0 | 0.7 | 11.1 | 6.5 | anoctamin-4 |
| Ano5 | 6.1 | 0.0 | 0.2 | 23.6 | 0.8 | 0.3 | 0.3 | anoctamin-5 |
| Ano6 | 4.8 | 1.6 | 19.4 | 8.2 | 2.1 | 20.8 | 21.0 | anoctamin-6 isoform 2 |
| Ano7 | 0.1 | 0.0 | 0.1 | 0.0 | 0.0 | 0.0 | 0.0 | anoctamin-7 |
| Ano8 | 12.1 | 1.2 | 9.0 | 5.2 | 0.2 | 14.3 | 10.9 | anoctamin-8 |
| Ano9 | 0.0 | 0.0 | 0.8 | 0.0 | 0.0 | 0.1 | 0.0 | anoctamin-9 |
| Ano10 | 14.1 | 6.1 | 13.7 | 18.3 | 5.6 | 18.7 | 20.0 | anoctamin-10 |
| Best1 | 0.5 | 0.0 | 0.7 | 0.4 | 107.9 | 0.4 | 0.3 | bestrophin-1 |
| Best2 | 0.0 | 0.0 | 0.0 | 0.0 | 0.0 | 0.0 | 0.1 | bestrophin-2 |
| Best3 | 0.8 | 0.0 | 0.1 | 2.7 | 4.0 | 1.4 | 0.3 | bestrophin-3 |
| Clcn1 | 8.4 | 0.0 | 0.2 | 33.2 | 0.1 | 0.1 | 0.5 | chloride channel protein 1 |
| Clcn2 | 8.9 | 7.9 | 4.6 | 0.8 | 3.2 | 12.9 | 8.1 | chloride channel protein 2 |
| Clca1 | 0.0 | 0.8 | 0.8 | 0.3 | 0.3 | 0.1 | 0.3 | chloride channel calcium activated 1 precursor |
| Clca2 | 0.3 | 0.2 | 4.1 | 0.3 | 0.0 | 0.5 | 0.5 | chloride channel calcium activated 2 precursor |
| Clca4 | 0.0 | 0.1 | 0.1 | 0.0 | 0.0 | 0.0 | 0.0 | chloride channel calcium activated 4 precursor |
| Cnp | 171.4 | 4.7 | 8.8 | 8.6 | 0.7 | 187.3 | 112.8 | 2',3'-cyclic-nucleotide 3'-phosphodiesterase isoform 1 |
| Gapdh | 5407.4 | 1030.4 | 788.7 | 10596.9 | 127.8 | 1132.6 | 1185.6 | glyceraldehyde-3-phosphate dehydrogenase |
| Hprt | 55.2 | 48.8 | 40.9 | 23.1 | 2.2 | 80.9 | 85.5 | hypoxanthine-guanine phosphoribosyltransferase |
| Ldha | 819.6 | 282.7 | 36.4 | 2373.2 | 186.6 | 135.8 | 192.8 | L-lactate dehydrogenase A chain isoform 2 |
| Ppia | 755.2 | 612.2 | 385.6 | 243.2 | 419.5 | 800.3 | 861.9 | peptidyl-prolyl cis-trans isomerase A |
| Rpl5 | 94.1 | 180.4 | 124.1 | 177.5 | 23.7 | 83.2 | 98.2 | 60S ribosomal protein L5 |
| Rpl19 | 379.1 | 290.9 | 199.1 | 303.6 | 38.0 | 147.0 | 159.0 | Rpl19 |
| Rpl29 | 252.7 | 211.6 | 127.0 | 248.1 | 219.9 | 96.9 | 122.0 | 60S ribosomal protein L29 |
| Tbp | 6.0 | 3.5 | 11.4 | 2.9 | 44.7 | 4.8 | 5.1 | TATA-box-binding protein |
| Trpa1 | 0.0 | 0.0 | 1.1 | 0.0 | 0.0 | 14.2 | 18.2 | transient receptor potential cation channel subfamily A member 1 |
| Trpm8 | 0.1 | 0.0 | 0.0 | 0.0 | 1.6 | 47.1 | 8.1 | transient receptor potential cation channel subfamily M member 8 |
| Trpv1 | 0.0 | 0.0 | 0.0 | 0.0 | 0.0 | 12.2 | 29.5 | transient receptor potential cation channel subfamily V member 1 |
| Ttyh1 | 134.8 | 0.2 | 8.0 | 0.6 | 5.7 | 31.2 | 27.2 | protein tweety homolog 1 isoform 1 |
| Ttyh2 | 15.4 | 15.4 | 4.1 | 4.5 | 4.6 | 10.3 | 12.2 | protein tweety homolog 2 |
| Ttyh3 | 58.1 | 3.2 | 26.4 | 1.9 | 7.2 | 13.2 | 10.8 | protein tweety homolog 3 |
| Tubb3 | 279.8 | 0.2 | 112.9 | 0.5 | 23.4 | 768.8 | 796.4 | tubulin beta-3 chain |
| Ubc | 411.4 | 403.1 | 236.5 | 417.3 | 234.1 | 212.1 | 221.1 | polyubiquitin-C |
